# Concurrent Training Decreases Cortisol but Not Zinc Concentrations: Effects of Distinct Exercise Protocols

**DOI:** 10.1155/2016/7643016

**Published:** 2016-04-05

**Authors:** Guilherme Rosa, Marcos de Sá Rego Fortes, Danielli B. de Mello

**Affiliations:** ^1^Research Group in Physical Exercise and Health Promotion, Physical Education Department, Castelo Branco University, Avenida Santa Cruz, 1631 Realengo, 21710-250 Rio de Janeiro, RJ, Brazil; ^2^Laboratory of Human Kinetics, The Federal University of State of Rio de Janeiro (LABIMH/UNIRIO), Brazil; ^3^Research Institute of Brazilian Army Physical Capacitation (IPCFEx), RJ, Brazil; ^4^Physical Education College of the Brazilian Army (EsEFEx), RJ, Brazil

## Abstract

*Objectives*. To investigate the effects of distinct concurrent training (CT) protocols on zinc and cortisol concentrations and test the correlation between these blood variables.* Methods*. Samples of serum zinc and cortisol were assessed from 10 male subjects (27.1 ± 4.8 years old; BMI 25.38 ± 0.09) before and immediately after each study session: control (CS = no exercises), concurrent training 1 (CT1 = indoor cycling + strength training), and concurrent training 2 (CT2 = strength training + indoor cycle) with five days of interval between each.* Results*. There were no significant changes in zinc concentrations after the CS (Δ% = 8.45; *p* = 0.07), CT1 (Δ% = 4.77; *p* = 0.49), and CT2 (Δ% = −2.90; *p* = 0.12) sessions. Cortisol levels showed significant decrease after CS (Δ% = −6.02; *p* = 0.00), CT1 (Δ% = −26.32; *p* = 0.02), and CT2 (Δ% = −33.57; *p* = 0.05) sessions. There was a significant correlation between the variables only at CS (zinc post versus cortisol pre: *r* = 0.82 and cortisol post: *r* = 0.82).* Conclusions*. CT decreases cortisol concentrations regardless of the sequence performed. No changes were found in zinc concentrations after the study sessions. The reduction in serum cortisol concentrations appear to occur by a mechanism independent of the zinc status.

## 1. Introduction

Zinc is a micronutrient involved in hundreds of reactions of cellular metabolism, including physiological processes such as growth and development, antioxidant defense, and immune function [1]. Therefore, a sufficient availability of this element is important for the immune system [2, 3]. For physically active individuals, adequate amounts of zinc in the diet are necessary to ensure increased energy expenditure and performance [4].

Brandão-Neto et al. [5] reported that both increased and decreased in serum zinc concentrations, resulting in changes in adrenal secretion. Cortisol is a glucocorticoid secreted by the adrenal cortex of the adrenal glands [6] which among other functions helps accelerate gluconeogenesis and the mobilization and utilization of fat to obtain energy, playing an important role both during and after exercise [7], also preventing the rupture of lysosomes, preventing further tissue degradation [8–10].

The effects of exercise on serum zinc levels seem to be dependent on the type, intensity, and duration of exercise [11]; however, studies [12, 13] demonstrated both decreased and increased serum zinc concentrations after physical exercise.

Regarding cortisol, investigations have been conducted aiming to verify the physical exercise effects on this hormone. As for zinc concentrations, some investigations [8, 14, 15] observed divergent responses between training protocols and research models.

For instance, Izquierdo et al. [16] demonstrated increased levels of cortisol in response to strength training, while Grandys et al. [17] did not observe significant changes after aerobic exercise. Dudgeon et al. [18] observed reduction in cortisol levels after aerobic and concurrent exercise protocols.

Most researchers who have investigated both zinc and cortisol concentrations in response to distinct physical exercise protocols used aerobic or strength exercises performed alone. The strategy that combines aerobic and strength exercises in the same session is known as concurrent training [19], commonly used to obtain the benefits of both strength training and aerobic exercise simultaneously [20, 21].

There is a lack of consensus of research that has been published on the effects of concurrent training on serum zinc and cortisol concentrations. Furthermore, investigations conducted aiming to test the correlation between zinc and cortisol concentrations in response to physical exercise are rare.

Therefore, the aim of this study was (a) to investigate the effects of distinct concurrent training protocols on serum zinc and cortisol concentrations in physically active adults and (b) test the correlation between these blood variables.

## 2. Methods

### 2.1. Subjects

The sample consisted of ten male volunteers (27.1 ± 4.8 years old, 74.89 ± 0.30 kg; 172 ± 0.03 cm; BMI 25.38 ± 0.09), who practiced regular aerobic and strength exercise for a minimum of six months, with weekly attendance of at least three days and no apparent risk factors that could prevent their participation in the study according to the Risk Stratification Criteria of the* American Heart Association (AHA)* [22].

The participants signed an informed consent document to participate in research involving human subjects in accordance with the Declaration of Helsinki [23]. The research project was also approved by the Ethics Committee in Research Involving Human Beings of the Universidade Castelo Branco (UCB, RJ, Brazil) under protocol number 0189/2008.

### 2.2. Data Collection

Body weight was measured using a 150 kg capacity scale with 100 g accuracy. Height was measured using a Filizola (Brazil) stadiometer. The aforementioned evaluations used the procedures recommended by the* International Society for the Advancement of Kinanthropometry (ISAK)* [24]. BMI was calculated through the ratio of body weight and the square of the height (kg/m^2^). At this time, participants answered the AHA/*American College of Sports Medicine* (ACSM) risk stratification questionnaire [22].

In the second phase of data collection, the One Repetition Maximum (1RM) test was performed [25], aimed at prescribing and controlling the intensity of the following exercises: supported rowing, 45° leg press, straight bench press, knee extensor, elbow extensor (HBM), knee flexor, and elbow flexor (high-pulley). Subjects also took part in an indoor cycling class, to familiarize themselves with the OMNI scale of perceived effort [26].

After the aforementioned procedures, all the 10 participants underwent 3 sessions: control session (CS), concurrent training 1 (CT1), and concurrent training 2 (CT2). The interval between each session was 5 days, during which subjects maintained normal sleep, food intake, and physical exercise routines.

### 2.3. Trials

#### 2.3.1. CS

Blood samples were collected from the participants to measure the baseline zinc and cortisol serum levels. Participants were required to fast for 12 hours and sleep for a minimum of eight hours, prior to blood draws. None of the participants performed any kind of physical exercise in the day before this session.

All blood collection procedures were taken at the study site by qualified nurses from “Sérgio Franco Medicina Diagnóstica” Laboratory, Brazil. A sample of blood (about 3 mL) was collected using mineral-free needles (25 × 0.7 mm), mineral-free vacuum tubes, and mineral-free proceeding gloves and transported to the laboratory for atomic absorption analysis to measure the serum zinc levels and chemiluminescence immunoenzymatic assay to measure serum cortisol concentrations.

Following the blood draw, participants had a breakfast that consisted of 200 mL fat-free yogurt, two slices of light whole wheat bread, 30 g of fresh white cheese, 10 g of margarine, and one medium-sized banana. Two hours after the first collection, new blood samples were collected to assess serum zinc and cortisol levels. Such procedures occurred between 6:30 am and 8:30 am.

#### 2.3.2. CT1 Session

This session occurred 5 days after the control session. Blood samples were collected following the same procedure adopted in the control session. Forty minutes after breakfast, the group held a concurrent training session composed by an indoor cycling class followed by a strength training session.

The indoor cycling class was continuously performed [9, 19, 27] and lasted about 40 minutes, divided as follows: warm-up of 5 minutes with an intensity between 2 and 4 of the OMNI scale of perceived exertion for cycling [26], continuous training of 30 minutes with an intensity between 5 and 7 (OMNI), and cooldown of 5 minutes with intensity between 0 and 2 (OMNI).

After cycling, the participants strength-trained. The strength training session was comprised of three sets of repetitions performed to exhaustion for each exercise in which the 1-RM was performed. The intensity was 85% 1-RM for all exercises and the rest interval between sets was two to three minutes. The order of the exercises was as described above.

#### 2.3.3. CT2 Session

In this session, the same procedures of the earlier sessions were followed, including the effort intensity; however, the concurrent training order was reversed: participants first conducted their strength training session followed by an indoor cycling class. In this session the strength training was preceded by five minutes warm-up on the treadmill, with an intensity ranging between 55% to 60% of hearth rate reserve (Karvonen method) [22] based on 220-age equation.

Immediately after sessions CT1 and CT2, participants' blood samples (same blood amount) were collected for analysis of the same blood variables. During the control session and concurrent training sessions participants were permitted to drink water (500 mL* ad libitum*) to avoid hemoconcentration.

After all blood analysis procedures, the remains of laboratory samples containing blood were discarded according to the ANVISA (Sanitary Vigilance National Agency of Brazil) legislation RDC 306 of December 7/2004, which deals with the technical regulation for waste management in health services.

### 2.4. Statistical Analysis

All of the statistical procedures were conducted using the* Statistical Package for the Social Sciences* software (SPSS 18.0, Chicago, USA). Descriptive statistics were used to establish the mean and standard deviation values. The Shapiro-Wilk (SW) test was used to verify the data normality. The two-way ANOVA was used for inferential analyses. Tukey's post hoc test was used to identify the possible differences. Pearson's correlation was used between the blood variables. A significance level of *p* < 0.05 was applied. A power analysis of the sample size was conducted.

## 3. Results

Two-way ANOVA found an interaction between the interventions sessions. The power of the experiment was of 98%, strengthening the magnitude of the results achieved in the analysis of the sample. It means that the sample size was enough to support the results.

Tables 1 and 2 show the results of the intra- and intergroups analysis of serum zinc and cortisol concentrations prior to (pre) and following (post) each of the respective sessions.

In the intragroup analysis, it was possible to observe that there was a tendency to increase in serum zinc concentration after CS and CT1 and a tendency to reduce the levels of the variable after CT2; however, these changes were not statistically significant. No significant difference between the study sessions was found.

Regarding the serum cortisol concentrations after the intragroup analysis, there was significant reduction after all sessions. However, the most expressive decreases were observed after CT1 and CT2 sessions. There was a significant decrease in cortisol after the intergroup analysis between CT2 versus CT1 and CT2 versus CS.

Table 3 shows Pearson's correlation between the blood variables at the pre and post moments of each session that comprised the study.

There was significant correlation between the blood variables only in the post moment of CS. This means that individuals who showed higher cortisol levels also had higher levels of zinc in the sample, because the correlation was positive.

## 4. Discussion

The present study aimed to investigate the effect of distinct exercise protocols characterized by concurrent training sessions on zinc and cortisol concentrations of physically active adults. The results demonstrate that despite the increase in zinc concentrations after CS and CT1 and the decrease after CT2 session, these changes were not statistically significant. However, cortisol levels showed a significant reduction in CS, CT1, and CT2 posttest.

Cordova and Alvarez-Mon [28] state that physical exercise can cause short-term effects on zinc levels and that these changes are dependent on the intensity. Although the intensity of the concurrent training sessions of this study might be considered moderate to elevated, there were no significant changes on participant's serum zinc concentrations.

The study conducted by Volpe et al. [29] demonstrated a significant reduction in zinc concentration after an aerobic exercise protocol. The authors assert that such reductions seem to reflect an acute stress response to strenuous exercise. This did not occur in the present investigation, where zinc levels did not change significantly after any of the sessions that made up the study.

González-Haro et al. [30] observed no significant changes in serum zinc concentrations after a single session of aerobic exercise with progressive intensity. The authors suggest that plasma volume maintained by adequate hydration has an important role on homeostasis of elements like zinc. This hypothesis might be supported by similar results obtained on this investigation, in which the subjects had water* ad libitum* and no significant changes in zinc levels were found.

The investigation of Simpson and Hoffman-Goetz [31] examined the effect of aerobic exercise sessions, held on a cycle ergometer, with different duration and intensity on serum concentrations of zinc in individuals with different levels of fitness. Their data demonstrate that, unlike in the present study, there were reductions in zinc levels of the subjects.

These data suggest that the effects of aerobic exercise on zinc concentrations are modified much more by the length than the intensity of exercise session. Furthermore, it seems that the level of physical fitness of individuals does not play a significant role on the responses of zinc status to physical exercise.

For Khaled et al. [32], zinc helps prevent the acute increase in blood viscosity induced by exercise, thereby improving the tolerance to that. In this light, the fact that there were no significant changes in concentrations of this variable after concurrent training sessions can be seen as positive.

Regarding cortisol, the other dependent variable of this investigation, there was significant decrease on the hormone concentration after the concurrent training sessions regardless of the sequence performed. These data are similar to the Kraemer et al. [15] study, which investigated the effects of strength training on hormonal responses.

According to Michailidis [10], the secretion of cortisol is related to stress. The exercise functioned as a stress factor, and the amount of hormone produced depends positively on the intensity and duration of exercise.

Rosa et al. [33] analyzed the acute effect of exercise on cortisol levels through a concurrent training session with similar characteristics of modes, volume, and intensity than the CT1 session of this study. Their results show that, as in the present study, the concurrent training protocol used induced a significant reduction on serum cortisol levels.

Comparing the serum cortisol responses to exercise, data of the study conducted by Izquierdo et al. [16] and the study of França et al. [8] differ from the present investigation results, showing significant increase. However, these authors used in their interventions strength and aerobic exercise protocols, respectively, performed alone, not concurrent training, explaining the divergent results.

Several hormones demonstrate circadian fluctuation and variation [34]. In some cases, these variations are due to regulatory endocrine axis pulses [35]; in others, they are related to humeral stimulus alterations caused by environmental or behavioral factors of the individual [36]. The influence of circadian cycle could explain the slight decrease on cortisol levels after CS [37].

Brandão-Neto et al. [5] reported that any change in zinc concentration can cause changes in adrenal secretion. As cortisol is secreted by adrenal cortex of adrenal glands, the secondary objective of this investigation was to test the correlation between zinc and cortisol responses to the exercise protocols.

In our study, despite changes in serum zinc levels on participant's blood were not statistically significant in any moment of the intervention, there was a significant decrease in serum cortisol levels. In addition, the correlation between the dependent variables was found only in CS.

The lack of correlation between the blood variables suggest that, at least after the concurrent training sessions with the characteristics used in this study, the reduction in cortisol concentrations occurred by a mechanism independent of the of zinc status.

## 5. Conclusions

Concurrent training decreases cortisol concentrations regardless of the sequence performed. The circadian cycle could explain the slight decrease in cortisol levels after CS. No changes were found in zinc concentrations after the study sessions. A significant correlation between the dependent variables was found only in CS.

Due to the relevance for both sports performance and health, further research on this subject is recommended with other exercise protocols, the biggest sample size, female participants, and individuals with distinct physical fitness status.

## Figures and Tables

**Table 1:** Analysis of serum *zinc (mcg/L)*.

	Pre	Post	Δ%	*p* value
CS	801.00 ± 87.49	868.70 ± 78.21	8.45	0.07
CT1	933.70 ± 115.55	978.30 ± 213.78	4.77	0.49
CT2	1302.10 ± 81.50	1264.30 ± 86.76	−2.90	0.12

CS: control session; CT1: concurrent training 1; CT2: concurrent training 2; Δ%: difference percent; *p* < 0.05.

**Table 2:** Analysis of serum *cortisol (mcg/dL).*

	Pre	Post	Δ%	*p* value
CS	13.94 ± 3.29	13.10 ± 3.17^#^	−6.02	0.00
CT1	18.61 ± 5.43	13.71 ± 4.87^#^	−26.32	0.02
CT2	14.98 ± 2.93	9.95 ± 2.26^#**∗**^	−33.57	0.05

CS: control session; CT1: concurrent training 1; CT2: concurrent training 2; Δ%: difference percent; #: significant difference (*p* < 0.05) within sessions; *∗*: significant difference (*p* < 0.05) between sessions (CT2 versus CT1 and versus CS).

**Table 3:** Correlation (*r*) between zinc and cortisol.

		CS		CT1		CT2	
		Zinc pre	Zinc post	Zinc pre	Zinc post	Zinc pre	Zinc post
Cortisol pre	*r*	0.56	0.82^§^	0.23	0.07	−0.04	0.00
	*p* value	*0.08*	*0.00*	*0.52*	*0.83*	*0.91*	*0.98*
Cortisol post	*r*	0.61	0.82^§^	−0.08	0.29	−0.00	0.18
	*p* value	*0.06*	*0.00*	*0.80*	*0.40*	*0.98*	*0.60*

§: correlation between the blood variables.
